# Glycine in Water Favors the Polyproline II State

**DOI:** 10.3390/biom10081121

**Published:** 2020-07-29

**Authors:** Brian Andrews, Shuting Zhang, Reinhard Schweitzer-Stenner, Brigita Urbanc

**Affiliations:** 1 Department of Physics, Drexel University, Philadelphia, PA 19104, USA; ba553@drexel.edu (B.A.); sz394@drexel.edu (S.Z.); 2 Department of Chemistry, Drexel University, Philadelphia, PA 19104, USA; rs344@drexel.edu

**Keywords:** molecular dynamics, protein folding, glycine

## Abstract

Conformational preferences of amino acid residues in water are determined by the backbone and side-chain properties. Alanine is known for its high polyproline II (pPII) propensity. The question of relative contributions of the backbone and side chain to the conformational preferences of alanine and other amino acid residues in water is not fully resolved. Because glycine lacks a heavy-atom side chain, glycine-based peptides can be used to examine to which extent the backbone properties affect the conformational space. Here, we use published spectroscopic data for the central glycine residue of cationic triglycine in water to demonstrate that its conformational space is dominated by the pPII state. We assess three commonly used molecular dynamics (MD) force fields with respect to their ability to capture the conformational preferences of the central glycine residue in triglycine. We show that pPII is the mesostate that enables the functional backbone groups of the central residue to form the most hydrogen bonds with water. Our results indicate that the pPII propensity of the central glycine in GGG is comparable to that of alanine in GAG, implying that the water-backbone hydrogen bonding is responsible for the high pPII content of these residues.

## 1. Introduction

The conformational manifolds of unfolded and intrinsically disordered peptides and proteins are many times described by the random coil model, which assumes that amino acid residues sample the entire sterically accessible space of the dihedral angles ϕ and ψ in the Ramachandran plot with comparable probabilities [1,2,3,4]. Deviations from the ideal random coil ensemble are generally believed to arise due to strong non-local interactions within compact or globular conformations or protein–solvent interactions in extended statistical coils [5,6,7]. However, this view has been modified over the last twenty years due to overwhelming experimental evidence which demonstrates that amino acid residues in a water sample a much more restricted space of the Ramachandran space than expected based on the above considerations. Many amino acid residues in unfolded protein regions exhibit significant intrinsic propensity for polyproline II (pPII) conformations [1,2,8,9,10,11,12,13,14]. In this context, pPII is understood as a local conformational state rather than the secondary structure of the entire or a significant portion of a disordered polypeptide chain [15,16]. Of all amino acid residues, alanine stands out by exhibiting a notoriously high pPII propensity with a mole fractions between 0.6 and 0.9 [14,17,18,19,20,21]. This observation and distinct pPII propensities of guest amino acid residues x in unblocked GxG peptides and corresponding blocked dipeptides [11,19,22,23,24] suggest that the pPII preference may be associated with the side-chain characteristics [19,22,25]. If this was the case, it would suggest that the side chains of amino acid residues and their respective conformations are primarily responsible for the observed reduction of the conformational backbone entropy [26]. If the conformational preferences of side chains dominated the Ramachandran plot of amino acid residues in water, the absence of a heavy-atom side chain in glycine would be expected to facilitate sampling of the entire sterically allowed region of the Ramachandran space, as originally predicted [1,2]. If, on the other hand, backbone hydration is the main force stabilizing the pPII state of alanine, as suggested by previous, mostly computational, studies which showed that, when in the pPII state, alanine residue accommodates water in a way that minimizes the electrostatic repulsion between peptide functional groups of the peptide and the mismatch between hydration and bulk water [27,28,29], then the conformational space of glycine residue should also be affected. Hence, determining the Ramachandran distribution of glycine in a polyglycine, which ensures the absence of nearest-neighbor interactions, is pivotal for the understanding how side chains and solvent affect the conformational distribution of the peptide/protein backbone.

Experimental studies on conformational ensembles of glycine residues are rare. Graf et al. explored the conformational space of cationic triglycine in water by MD simulations constrained by a set of seven J-coupling constants, suggesting a rugged free energy landscape with a large number of minima distributed all over the Ramachandran space [18]. The results of this study are in line with the hypothesis of a rather unrestricted conformational distribution in the Ramachandran space. However, quantum mechanical (QM) calculations on glycine-containing peptides, which are often used to calibrate molecular dynamics (MD) force fields [30,31], and comprehensive analysis of resolved protein structures [32] suggest that even glycine samples a rather restricted conformational space. Understanding conformational dynamics of glycine in water is important because a comparison of the conformational ensemble of glycine to those of other amino acids can provide insights into the interdependence of the side chain and backbone solvation, which has implications for protein folding theories [33,34,35].

Because some MD force fields are based on dihedral potentials of short alanine-based as well as glycine-based peptides, their accuracy depends on how well these potentials capture intrinsic conformational dynamics of amino acid residues [36,37]. Moreover, a clear assessment of the effect of the side chain versus backbone structure and solvation on conformational preferences of amino acid residues in water requires a determination of the intrinsic conformational propensities of the backbone as a reference model system. In this paper, we analyze the published spectroscopic data: NMR coupling constants [18] and amide I’ IR and Raman profiles [38] to derive the Ramachandran distribution of the central glycine in cationic triglycine in water that best reproduces all available experimental data using the Gaussian model method recently applied to alanine [39]. Including amide I’ band profiles in addition to the J-coupling constants is important because these profiles are sensitive to conformational sampling due to the strong nearest-neighbor coupling between the respective normal modes of peptide groups which lead to a significant non-coincidence between the peak positions of IR, isotropic, and anisotropic Raman profiles [40,41,42]. These combined experimental data enable the construction of an experiment-based Ramachandran distribution of the central glycine in cationic triglycine in water, which is referred hereafter as the Gaussian model distribution, offering a reference for the assessment of the three commonly used MD force fields with respect to their ability to reproduce the experimental data for the central glycine in triglycine.

## 2. Materials and Methods

### 2.1. Molecular Dynamics Simulations

#### 2.1.1. Simulations of Tripeptides in Water

Tripeptides GGG, GAG, AAA, and GPG were constructed using the Visual Molecular Dynamics (VMD) software package [43]. A single tripeptide was immersed into a 64 nm${}^{3}$ cubic box with periodic boundary conditions at temperature 300 K using GROMACS 5.1.2 [44,45,46,47,48,49,50]. The following three combinations of force fields and water models were used: Amber ff14SB [37] with TIP3P [51], OPLS-AA/M [36] with TIP4P [51], and CHARMM36m [52,53,54,55] with TIP3P. In each simulation, the tripeptide under consideration was protonated at the N terminus (NH${}_{3}^{+}$) and neutral at the C terminus to mimic acidic pH used in experiments. When using OPLS-AA/M and CHARMM36m, the C terminus was capped with a carboxyl group (COOH), whereas an amino group (CONH${}_{2}$) capping of the C terminus was applied in Amber ff14SB. A single Cl${}^{-}$ ion was added to obtain an electrostatically neutral system. The Verlet cutoff scheme [56] and a time step of 2 fs were used during the equilibration and production steps. The steepest descent was utilized for energy minimization for 100,000 time steps, followed by a 20 ps pressure equilibration step at 300 K and 1.0 bar. Production runs using the velocity rescale thermostat [57] and Berendsen barostat [58] resulted in 300 ns-long trajectories. All Ramachanran distributions are calculated within GROMACS 5.1.2 using time frames (separated by 2ps) within 50–300 ns of each MD trajectory. Additional simulations of GGG were performed with NH${}_{3}^{+}$ and COO${}^{-}$ cappings at the N and C termini, respectively, to determine the effects of end groups on the analysis. These simulations were prepared under the same protocol as mentioned above aside from the addition of ions, which is redundant as the peptide is neutral.

#### 2.1.2. Simulations of Triglycine in Dimethyl Sulfoxide

GGG was also simulated in a cubic box solvated by dimethyl sulfoxide (DMSO) using Amber ff14SB and CHARMM36m. A structure file of DMSO retrieved from the ZINC database [59] was used for simulations with Amber ff14SB and CHARMM36m. For simulations with Amber ff14SB, parameters for DMSO were derived using the General Amber Force Field (the Leap tool in AmberTools19 [60]). Antechamber Python parser interface (ACPYPE) [61] was then used to convert the AMBER parameter files to GROMACS-compatible files. For CHARMM36m, SwissParam software [62] was used to derive parameters for DMSO. In each simulation, a cubic box of 64 nm${}^{3}$ was filled with 438 DMSO molecules, corresponding to 11.36 M concentration. All other preparation, including N and C terminal groups, followed the same protocol as for simulations in pure water described above.

#### 2.1.3. Simulations of Triglycine in Tetrachloride

GGG was also simulated in a cubic box solvated by carbon tetrachloride (CCl${}_{4}$) using Amber ff14SB and CHARMM36m. A PDB file for the CCl${}_{4}$ structure was created within VMD according to data from the NIST database [63] for use with Amber ff14SB. A structure file of CCl${}_{4}$ retrieved from the ZINC database [59] was used for simulations with CHARMM36m. All bond lengths and bond angles were set to 1.74 Å and 109${}^{\circ }$, respectively. For simulations with Amber ff14SB, parameters for CCl${}_{4}$ were derived using the General Amber Force Field (the Leap tool in AmberTools19 [60]). An antechamber Python parser interface (ACPYPE) [61] was then used to convert the AMBER parameter files to GROMACS-compatible files. For CHARMM36m, SwissParam software [62] was used to derive parameters for CCl${}_{4}$. In each simulation, a cubic box of 64 nm${}^{3}$ was filled with 400 CCl${}_{4}$ molecules, corresponding to 10.3 M concentration. In this case, the N and C termini of GGG were neutral. In Amber ff14SB, the N terminus was capped by an acetyl group (-C(=O)-CH${}_{3}$) and the C terminus was capped by an amino group. In CHARMM36m, the N and C termini were capped by an amino and carboxyl group, respectively. All other preparation followed the same protocol as for simulations in pure water described above.

### 2.2. Analysis

#### 2.2.1. J-Coupling Constants, Amide I’ Profiles, Gaussian Model, and $\chi^{2}$ Functions

The experimental data for the central glycine in cationic GGG reported by Graf et al. include five J-coupling constants: ${}^{3}J\left(H^{N},H^{C_{\alpha }}\right)$, ${}^{3}J\left(H^{N},C^{\prime }\right)$, ${}^{3}J\left(C,C^{\prime }\right)$, ${}^{3}J\left(H^{C_{\alpha }},C^{\prime }\right)$, and ${}^{1}J\left(N,C_{\alpha }\right)$ [18]. Due to achiral nature of triglycine, neither amide I’ nor any other vibrational mode exhibits any dichroism [38]. Here, we use the Gaussian model, previously developed by Schweitzer-Stenner and colleagues in order to construct the model Ramachandran distribution for the guest amino acid residue in tripeptides from the available J-coupling constants and amide I’ profiles [64]. The Gaussian model describes conformational distributions of individual residues as a superposition of statistically weighted two-dimensional Gaussian distributions associated with known secondary structures. Statistical weights, peak positions, and halfwidths of these distributions are adjustable parameters for the reproduction of experimental data. The statistical weights are expressed in mole fractions and represent the propensities of an amino acid residue for a given secondary structure. As described previously for alanine in GAG and AAA [39], MD-derived and Gaussian Ramachandran distributions of the central glycine in cationic GGG are used to calculate (a) the J-coupling constants using Karplus equations [18] with Karplus parameters and their uncertainties, previously derived from X-ray and solution data [65,66], and (b) amide I’ profiles, which we calculated by utilizing the orientational dependence of excitonic coupling between the two amide I’ modes of the investigated tripeptides [64]. For the construction of the Ramachandran plots, we subdivided the Ramachandran space into 32,400 bins with a size $2^{\circ }\times 2^{\circ }$ for which we calculated occurrence probabilities.

To quantitatively assess the ability of MD force fields and the Gaussian model to capture the NMR data on the central glycine in cationic GGG, we use a reduced $\chi_{J}^{2}$ function:

(1) $$\begin{array}{ccc} \chi_{J}^{2} & = & \frac{1}{N}\sum_{i=1}^{N}\frac{{\left(J_{i,exp}-J_{i,calc}\right)}^{2}}{s_{i}^{2}} \end{array}$$

where *N* is the number of J-coupling constants (in our case five), $J_{i,exp}$ are the experimental J-coupling constants, $J_{i,calc}$ are the calculated constants calculated from the dihedral angle distribution (obtained by MD or from the Gaussian model), and $s_{i}$ are the uncertainties derived from the reported experimental errors [18], and the errors associated with the Karplus parameters [66] using Gaussian error propagation.

By construction, the Gaussian model for the central glycine in GGG takes into consideration the achiral nature of GGG and is thus invariant to the (ϕ, ψ) to (−ϕ, −ψ) transformation, resulting in a zero VCD amide I’ profile. However, MD-derived Ramachandran distributions may not be completely invariant to this transformation due to limited sampling or for other reasons. We thus assess the three MD force fields under investigation with respect to their ability to reproduce the absence of the amide I’ signal by calculating the $\chi_{VCD}^{2}$ function:

(2) $$\begin{array}{ccc} \chi_{VCD}^{2} & = & \frac{1}{N}\sum_{i=1}^{N}{\left(\Delta \epsilon_{exp,k}-\Delta \epsilon_{calc,k}\right)}^{2} \end{array}$$

where *N* is the number of wavenumbers from 1600 to 1720 cm${}^{-1}$ and $\Delta \epsilon_{exp,k}$ is set to zero for all *k*. Note that experimental data for the central glycine in cationic GGG cannot be obtained because the experimental error is larger than the measured signal.

#### 2.2.2. Definition of Mesostates

Mesostates are used to compare conformational distributions derived from experimental data and MD simulations. The following mesostate definitions are used: (a) pPII ($-90^{\circ }<\phi <-42^{\circ }$, $100^{\circ }<\psi <180^{\circ }$), (b) anti-parallel β-strand (aβ) ($-180^{\circ }<\phi <-130^{\circ }$, $130^{\circ }<\psi <180^{\circ }$), (c) the transition region between aβ and pPII (βt) ($-130^{\circ }<\phi <-90^{\circ }$, $130^{\circ }<\psi <180^{\circ }$), (d) right-handed α-helix ($-90^{\circ }<\phi <-32^{\circ }$, $-60^{\circ }<\psi <-14^{\circ }$). The mesostate populations are calculated from the MD trajectories using time frames 50–300 ns as the number of conformations within each mesostate region normalized by the total number of conformations. Because triglycine is achiral, the (−ϕ,−ψ) conformations are identical to the corresponding (ϕ,ψ) conformations. Consequently, for the central glycine in triglycine, the mesostate populations are obtained by adding the respective left-handed and right-handed populations.

#### 2.2.3. Shannon Entropy

The Ramachandran distributions produced by the Gaussian model and MD force fields are used to calculate the Shannon entropy for the central glycine residue in GGG and alanine residue in GAG as follows:

(3) $$\begin{array}{ccc} S & = & -R\sum_{\phi ,\psi }P\left(\phi ,\psi \right)\;\ln P\left(\phi ,\psi \right) \end{array}$$

where *R* is the ideal gas constant and P(ϕ,ψ) is the probability distribution of the backbone dihedral angles ϕ and ψ of the guest residue.

#### 2.2.4. Water Orientation Plots

Water orientation plots, which were introduced by Meral et al. [25], were applied here to probe the average water orientation adjacent to the backbone of the central amino acid in cationic tripeptides GGG, GAG, AAA, and GPG. Using VMD, we extract the orientation of each water molecule in a 4 Å-thick hydration layer around the backbone atoms of the amino acid under investigation. The orientation of each water molecule is characterized by angles η and θ, defined relative to the normal to the solvent accessible surface (SAS), $\hat{n}$. η is the angle between the water symmetry axis and $\hat{n}$ and θ is the angle between the normal to the water plane (defined by the centers of mass of water’s oxygen and the two hydrogens) and $\hat{n}$. A 2D histogram in the (η,θ) space ($\eta \in [0^{\circ },180^{\circ }]$ and $\theta \in [0^{\circ },90^{\circ }]$) is created with 8100 bins (90 × 90). The histogram, which is calculated using time frames within 250–300 ns of each MD trajectory under consideration, displays a probability distribution of water orientations within the hydration layer adjacent to the backbone of the central amino acid. We also calculated the average probability of water to adopt orientations within the region (η, θ) space: ($100^{\circ }<\eta <140^{\circ }$, $67^{\circ }<\theta <90^{\circ }$) by summing over normalized values of all probability density bins within this region. To estimate the error, we derived water orientation plots from 500 conformations (2 ps apart) within 1 ns long window (250–251 ns, 251–252 ns, ⋯ 299–300 ns) of each trajectory under consideration. For each of these individual water orientation plots, we calculated the average and the standard error of the mean (SEM) of the probability that water adopts the above orientation, in which the normal to the water plane is approximately perpendicular to $\hat{n}$ and one of the water hydrogens points toward the SAS, i.e., water orientations associated with water forming hydrogen bonds (HBs) with the functional backbone groups.

#### 2.2.5. Solvent-Peptide and Intrapeptide HBs

The HB is defined within GROMACS 5.1.2 by the donor–acceptor distance smaller than or equal to 3 Å and the angle between the donor, hydrogen, and acceptor within 20${}^{\circ }$. The average number of HBs between water and the functional backbone groups of the central residue in GGG, GAG, AAA, and GPG was calculated and the respective SEM values were computed from per-nanosecond averages using time frames within the 250–300 ns of each trajectory under consideration. This analysis was also applied to simulations of GGG in DMSO. In the case of a nonpolar solvent CCl${}_{4}$, the average number of intrapeptide HBs was calculated using the same HB parameters.

## 3. Results

### 3.1. Experiment-Based Ramachandran Distribution for the Central Glycine in GGG Reveals High PPII Content

**Figure 1:**
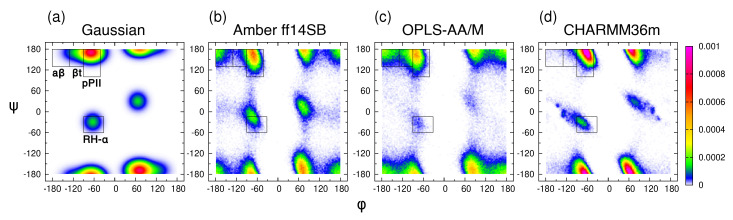
Ramachandran distributions of the central glycine in GGG from (**a**) the Gaussian model and MD with (**b**) Amber ff14SB, (**c**) OPLS-AA/M, and (**d**) CHARMM36m. The rectangular boxes correspond to the four mesostates.

The experimental data on the central glycine residue in cationic GGG in water include five J-coupling constants, ${}^{3}J\left(H^{N},H^{C_{\alpha }}\right)$, ${}^{3}J\left(H^{N},C^{\prime }\right)$, ${}^{3}J\left(C,C^{\prime }\right)$, ${}^{3}J\left(H^{C_{\alpha }},C^{\prime }\right)$, and ${}^{1}J\left(N,C_{\alpha }\right)$ [18], and amide I’ profiles obtained using IR and Raman spectroscopy [38]. As in the previous study [39], we introduce four mesostates: pPII, βt, aβ, and α, corresponding to specific regions in the Ramachandran space (Figure 1a). We then apply the Gaussian model, in which the pPII, β-strand, and α-helical states are modeled as Gaussian sub-distributions in the (ϕ, ψ) space with the weights, locations, and widths optimized to best fit the experimental data [64]. Briefly, the Gaussian model for glycine is comprised of three Gaussian sub-distributions corresponding to pPII, β, and right-handed helical mesostates alongside the corresponding sub-distributions on the right side of the Ramachandran space due to nonchiral nature of glycine reflected in the inversion symmetry of the Ramachandran distribution. This selection automatically accounts for some turn-like conformations. In a first step, we use this three/six state Gaussian model to reproduce the experimental data. The ϕ and ψ coordinates of the pPII and β sub-distributions are moved within certain intervals defined by the boundaries of the respective mesostate (for pPII: $-80^{\circ }<\phi <-65^{\circ }$ and $140^{\circ }<\psi <170^{\circ }$; for β-strand: $-140^{\circ }<\phi <-100^{\circ }$ and $140^{\circ }<\psi <170^{\circ }$ on the left-hand side of the Ramachandran space). In line with the earlier application of the Gaussian model [19], the coordinates for the sub-distributions of right-handed helical conformers are set to $\phi =-60^{\circ }$ and $\psi =-30^{\circ }$ and allowed to slightly vary along the ϕ-coordinate alongside their symmetric sub-distribution counterparts on the right-hand side of the Ramachandran space. The search for the best fit is guided by the Karplus curves of the J-coupling constants described above. Once the best global fit to the experimental data was obtained, we added a small fraction of γ-turns (and their symmetric counterparts) to test whether or not this addition would improve the fit. This change resulted in insignificant changes of the calculated J-coupling constants and amide I’ profiles, indicating that the three/six state Gaussian model produces the best fit to experimental data. This solution of the Gaussian model for the central glycine in GGG is shown in Figure 1a and is hereafter referred to as the Gaussian Ramachandran distribution or plot.

Table S1 shows that the J-coupling constants derived from the Gaussian Ramachandran distribution are in a good agreement with the respective experimental values for ${}^{3}J\left(H^{N},H^{C_{\alpha }}\right)$, ${}^{3}J\left(H^{N},C’\right)$ and ${}^{3}J\left(H^{C_{\alpha }},C^{\prime }\right)$. For ${}^{3}J\left(C,C’\right)$, the difference between the calculated and experimental value is somewhat larger, possibly due to unknown uncertainties of the Karplus parameters for this coupling constant, which complicates the assessment of the deviation. A similar discrepancy between experimental and computational values of this coupling constant has been observed for trialanine [18,20]. It should be noted that the empirical Karplus plots always produce positive values for the respective J-coupling constant [39], while it can become negative in reality. However, such negative coupling values cannot be discerned from conventional one- and two-dimensional NMR experiments. This could lead to overestimated value of a J-coupling constant if this value is close to a minimum in the Karplus plot and if the respective minimal value is close to or equal to zero. It is noteworthy that the calculated ${}^{1}J\left(N,C_{\alpha }\right)$ underestimates the experimental value, which is significantly higher than corresponding values for, e.g., the central alanine in GAG or AAA [18,19]. This observation is clearly indicative of a very large average ψ-value of the conformational ensemble (*vide infra*). The above calculation of J-coupling constants followed the study by Graf et al. [18] who used the Karplus parameters of Wirmer and Schwalbe [67]. We asked to which extent the selection of Karplus parameters might affect the comparison of the calculated and experimental ${}^{1}J\left(N,C_{\alpha }\right)$. To this end, we employed a slightly different parameter set reported by Ding and Gronenborn [68], which yielded a higher value (12.1 Hz) of ${}^{1}J\left(N,C_{\alpha }\right)$ that is very close to the experimental value (Table S1). The amide I’ profiles in Figure S1 show the experimental IR and Raman profiles of the amide I’ mode alongside respective quantities calculated from the Gaussian Ramachandran distribution. The calculated isotropic Raman scattering profile is in a good agreement with the experimental counterpart. This is important because the strong asymmetry of the band profile reflects the degree of excitonic coupling between the two amide I’ modes [69]. The calculated anisotropic Raman and IR profiles somewhat overestimate the lower and higher wavenumber band, respectively.

The Gaussian Ramachandran distribution for the central glycine (Figure 1a) reveals that its conformational space is concentrated predominantly within the broader pPII region, split between the left- and right-handed counterparts. Significantly lower populations are found in the βt and helical regions, whereas the aβ region is only sparsely populated. The mesostate populations for glycine in achiral GGG (Table S2) corresponds to the sum of the left-handed and right-handed counterparts. A comparison of the pPII basins of the central glycine and alanine residues in GxG demonstrates that the population of left-handed pPII conformations for glycine is shifted to larger ψ values (compare Figure 1 to Figure 1a in Zhang et al. [39]), indicating that the $C_{\beta }$ carbon group of alanine modifies the pPII conformation relative to that of glycine. Consequently, a significant fraction of glycine’s pPII conformations falls outside of the pPII mesostate as initially defined for alanine. Shifting the pPII and both β mesostate regions by 31${}^{\circ }$ and 16${}^{\circ }$, respectively, in the positive ψ (while keeping the area of each region intact) results in increased pPII and βt populations for the Gaussian model of glycine in GGG, on par with mesostate populations of alanine in GAG (Table S2). Consequently, the orientational angle between the two peptide groups of the central glycine in GGG is shifted from ∼80${}^{\circ }$ (ψ≈ 150${}^{\circ }$) to ∼70${}^{\circ }$ (ψ ≈ 170${}^{\circ }$), thereby making pPII conformations of glycine in GGG more extended than the respective alanine conformations in GAG. These findings demonstrate that the population of the pPII state, albeit modified relative to that of alanine, dominates the conformational space of glycine.

### 3.2. Conformational Ensembles of the Central Glycine in GGG: Assessment of MD Force Fields

We next performed MD simulations of cationic GGG in water (see Section 2.1. for details) using three commonly used MD force fields: Amber ff14SB (with TIP3P water), OPLS-AA/M (with TIP4P water), and CHARMM36m (with TIP3P water). Amber ff14SB and CHARMM36m were both developed alongside their respective TIP3P water models, whereas no specific water model was used in OPLS-AA/M parameterization, so our selection of TIP4P water is based on the comparison of water models reported in our previous study which revealed a relatively modest effect of the water model on conformational dynamics of the central alanine in GAG and AAA [39]. Hereafter, the term ’force field’ is used to include also the force field-specific water model parameters. The Ramachandran distributions of the central glycine in GGG for the three force fields in Figure 1b–d showcase force field-specific features that deviate from the predictions of the Gaussian model. For all three force fields, the pPII basin is shifted to higher ψ values relative to the pPII basin of alanine in GAG, consistent with the predictions of the Gaussian model. Amber ff14SB and OPLS-AA/M predict an increased population within and left of the aβ mesostate region and its left-handed counterpart, not present in the Gaussian model (Figure 1a–c). All mesostate populations are reported in Table S2.

**Figure 2:**
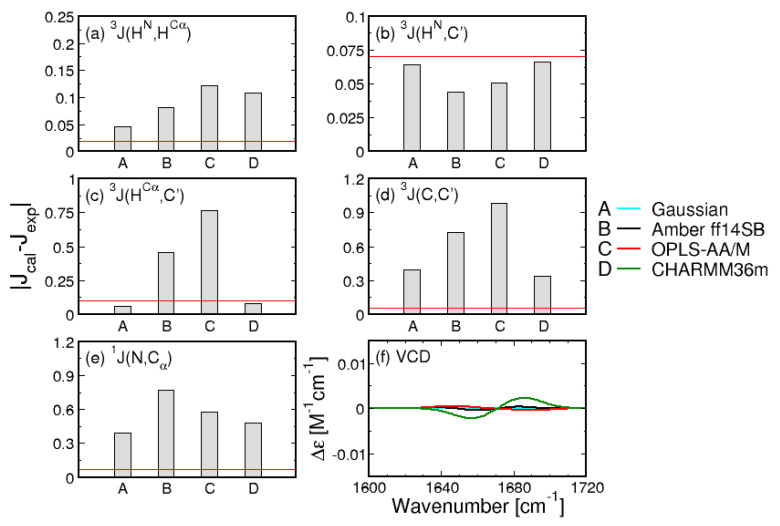
Comparison between experimental and calculated J-coupling constants. (**a**–**e**) absolute differences between calculated and experimental values of the five J-coupling constants for the Gaussian model and the three MD force fields. Red lines correspond to experimental uncertainties; (**f**) amide I’ profiles calculated from MD-derived Ramachandran distributions.

While the comparison of Ramachandran plots in Figure 1 is visually compelling, it relies on the somewhat arbitrary definition of mesostates. To quantitatively assess the Gaussian model and the three MD force fields, the entire Ramachandran distributions in Figure 1 are used to calculate the five J-coupling constants and amide I’ profiles, providing a comparison independent of the definition of mesostates. The absolute differences between the calculated and experimental J-coupling constants (Figure 2a–e) and MD-derived amide I’ profiles (Figure 2f) indicate that the Gaussian model outperforms MD force fields for four out of five J-coupling constants (Figure 2a–e). Only in the case of ${}^{3}$J(C,C’), CHARMM36m reproduces the experimental value better than the Gaussian model. The five J-coupling constants were also calculated for all 50 ns-long intervals demonstrating only minor fluctuations over the course of the trajectories (Figure S2). Figure S1 demonstrates that the conformational distributions produced by Amber ff14SB and OPLS-AA/M reproduce the amide I’ profiles as well as the Gaussian model, whereas the corresponding profiles produced by CHARMM36m exhibit more pronounced deviations from experimental data.

**Figure 3:**
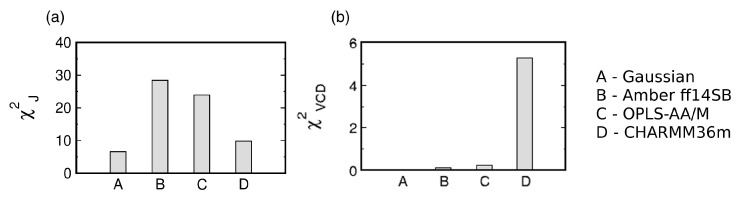
Assessment of the Gaussian model and three MD force fields with respect to their ability to reproduce the experimental data for the central glycine residue in cationic GGG by (**a**) $\chi_{J}^{2}$ and (**b**) $\chi_{VCD}^{2}$. $\chi_{VCD}^{2}$ values in (**b**) are multiplied by $10^{7}$ for display purposes.

The three MD force fields and the Gaussian model can be assessed by considering the two $\chi^{2}$ functions (see Section 2.2. for details). Figure 3 shows the reduced $\chi_{J}^{2}$ and $\chi_{VCD}^{2}$ values for the Gaussian model and the three MD force fields. With regard to $\chi_{J}^{2}$, CHARMM36m outperforms OPLS-AA/M. Amber ff14SB ranks the lowest of the three force fields. In contrast, CHARMM36m ranks the lowest in its capability to reproduce the experimental amide I’ profiles as reflected in $\chi_{VCD}^{2}$ values. In order to account for the achiral nature of triglycine, the Ramachandran plot of the Gaussian model was set up so that the conformational distribution was symmetric with regard to the inversion center at ϕ=ψ=0. As a consequence, the ensemble average of the rotational strength of amide I’ was set to zero. On the contrary, MD-derived Ramachandran distributions possess some degree of asymmetry due to unequal sampling of the right- and left-handed conformations, resulting in nonzero rotational strengths of amide I’. Figure S3 demonstrates that, for Amber ff14SB and OPLS-AA/M, the amide I’ VCD signal is low and displays small fluctuations as the sampling time is increased (Δ$\chi_{VCD}^{2}$<$10^{-7}$). For CHARMM36m, the amide I’ VCD signal significantly decreases upon increased sampling but the final signal, although below the experimentally detectable value, is not as low as for the other two force fields. CHARMM36m [55] and its predecessor CHARMM36 [54] are based on CHARMM22 [52] with additional modifications, including empirical CMAP corrections [70]. These CMAP corrections are based on the resolved protein structures in crystal environments and may induce a degree of chirality as well as correlation between ϕ and ψ due to the local guest residue neighborhood. This is consistent with a recent study on a large number of glycine residues from crystal protein structure showing that glycine adopts chiral nature reflected in an asymmetric Ramachandran distribution when embedded in a chiral environment [71]. Empirical CMAP corrections in CHARMM36m may have introduced a degree of chirality into the empirical potential for glycine reflected in the inexact balance between left- and right-handed conformations and a nonzero VCD signal. Overall, the comparison to experimental data shows that the Gaussian model outperforms all three MD force fields, whereas the comparison among the three MD force fields is less straightforward: CHARMM36m outperforms the other two force fields with respect to J-coupling constants; however, if both $\chi^{2}$ functions are considered, OPLS-AA/M shows a better agreement with experimental data than the other two force fields. We also examined the effect of neutral versus negatively charged C terminus of GGG on the conformational ensemble of central glycine for all three MD force fields to demonstrate that this capping does not exert any significant effect (Figures S4 and S5 in Supplementary Materials).

Two additional differences between the Gaussian and MD-derived Ramachandran distributions of the central glycine in GGG are worth discussing. First, the pPII and helical basins are more asymmetric in the MD-derived than in the Gaussian Ramachandran distribution. The Gaussian model solution for glycine residue is associated with slightly asymmetric Gaussian sub-distributions for pPII and β states with smaller standard deviations for ψ (15${}^{\circ }$) than for ϕ (20${}^{\circ }$). The MD-derived Ramachandran distributions, however, indicate a substantially wider basins along the ψ-coordinate. We thus asked to which extent an increase of the width of the pPII and β sub-distributions along the ψ-coordinate in the Gaussian model would affect the J-coupling constants and amide I’ profiles. We modified the Gaussian model solution by increasing the width from 15${}^{\circ }$ to 25${}^{\circ }$ along the ψ-coordinate in both pPII and β sub-distributions, which resulted in a larger difference between the calculated and experimental value for ${}^{1}J\left(N,C_{\alpha }\right)$ (data not shown), demonstrating that increasing the widths of the two basins along the ψ-coordinate makes the Gaussian model less consistent with experimental data. Second, in MD-derived (but not the Gaussian) Ramachandran distributions, the pPII and β basins are narrow and slanted with respect to the ϕ- and ψ-coordinates, indicative of negative correlations. We asked whether or not introducing such correlations into the Gaussian model would affect the resulting J-coupling constants. To address this question, we modified the Gaussian model by rotating the axes of the basins counterclockwise, assuming negative off-diagonal correlations, producing the asymmetric shapes that are particularly notable in the Ramachandran distribution obtained with CHARMM36m. Our results indicate that this manipulation had no effect on the calculated J-coupling constants and amide I’ profiles (data not shown), demonstrating that the available experimental data are insensitive to potential ϕ−ψ correlations.

Another way to compare MD-derived Ramachandran distributions of guest residues to the Gaussian model counterparts is through the Shannon entropy associated with the distribution (see Section 2.2. for details). The difference between the Shannon entropy of each MD-derived and Gaussian Ramachandran distribution, $\Delta S_{I}$, is reported in Table S3 (row 1) for each of the three MD force fields. The largest and positive value of $\Delta S_{I}$ is associated with OPLS-AA/M ($5.07\;J\;{\mathrm{mol}}^{-1}\;K^{-1}$), a smaller and positive $\Delta S_{I}$ is predicted by Amber ff14SB, whereas CHARMM36m displays the lowest and negative $\Delta S_{I}$. A similar comparison for alanine in GAG (Table S3, row 2) using previously published Ramachandran distributions (see Table S2 in Zhang et al. [39]) reveals a similar trend: OPLS-AA/M predicts the largest $\Delta S_{I}$ value of ($1.49\;J\;{\mathrm{mol}}^{-1}\;K^{-1}$), followed by Amber ff14SB, whereas CHARMM36m is again associated with the smallest and negative $\Delta S_{I}$. Notably, Amber ff14SB results in a significantly lower $\Delta S_{I}$ for alanine in GAG than for the central glycine in GGG, consistent with a much better performance of this force fields with respect to alanine conformational ensembles [39].

If the difference between the central glycine in GGG and alanine in GAG stemmed only from the achiral nature of the former, one would expect the difference between the Shannon entropies of the central glycine in GGG and alanine in GAG to be $\Delta S_{II}=R\;\ln2=5.76\;J\;{\mathrm{mol}}^{-1}\;K^{-1}$. The $\Delta S_{II}$ value calculated from the Gaussian Ramachandran distributions of the central glycine in GGG and alanine in GAG is $3.24\;J\;{\mathrm{mol}}^{-1}\;K^{-1}$ (Table S3, row 3), which is significantly lower than the expected value. The corresponding $\Delta S_{II}$ values for Amber ff14SB ($7.48\;J\;{\mathrm{mol}}^{-1}\;K^{-1}$), and OPLS-AA/M ($6.81\;J\;{\mathrm{mol}}^{-1}\;K^{-1}$) are closer to the expected value, whereas CHARMM36m is associated with the overall lowest value of $2.41\;J\;{\mathrm{mol}}^{-1}\;K^{-1}$, which is the closest to the Gaussian model prediction. Such a low value of $\Delta S_{II}$ (relative to the expected value) suggests that embedding glycine into a chiral environment would increase the local conformational entropy less than embedding alanine, which is a counterintuitive result.

### 3.3. The PPII State Enables Glycine and Alanine Residues to Form the Most HBs with Water

**Figure 4:**
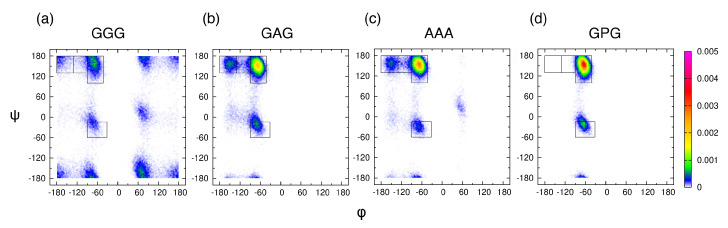
Ramachandran distributions for the guest residue in (a) GGG, (b) GAG, (c) AAA, and (d) GPG derived from MD simulations in Amber ff14SB and TIP3P using conformations within 50–300 ns of each trajectory. The rectangular boxes correspond to the four mesostates (see Figure 1a).

Examining the central alanine in GAG and AAA, Zhang et al. showed that, of the four mesostates, pPII is associated with, on average, the most HBs between water and alanine residue [39]. We here ask whether or not the average orientation of water in the hydration layer, adjacent to the backbone atoms of the guest residue, is affected by its propensity for the pPII state. We selected four tripeptides with glycine, alanine, and proline as guest residues and performed MD simulations with Amber ff14SB. Ramachandran distributions in Figure 4 indicate that the pPII population of the guest residue progressively increases in the order of GGG < GAG < AAA < GPG.

**Figure 5:**
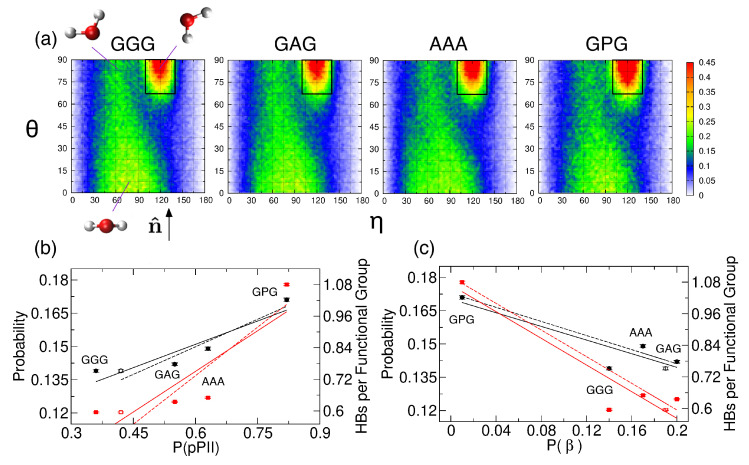
(**a**) Water orientation plots for hydration water adjacent to the backbone of the guest residue of GGG, GAG, AAA, and GPG. The leftmost plot shows three characteristic water orientations with respect to the normal $\hat{n}$ to the SAS. The black rectangular region, defined in Section 2.2., contains the most populated water orientations. The color corresponds to the probability density (with scale values multiplied by 10${}^{3}$). (**b**,**c**) The probability of the most populated water orientation (black solid circles) and the average number of water-guest residue HBs normalized by the number of functional groups (red solid squares) are shown as a function of the (**b**) pPII and (**c**) β populations. The solid black and red lines in (**b**) are a result of a linear regression analysis with Pearson’s r=0.92 (the probability) and r=0.86 (the average number of HBs). The solid black and red lines in (**c**) show a result of the regression analysis with r=−0.89 (the probability) and r=−0.93 (the average number of HBs). Black and red dashed lines with error bars correspond to the regression analysis performed by using the shifted pPII and β populations for the central glycine residue in GGG (black open circles and red open squares in **b** and **c**, respectively). The dashed black and red lines in (**b**) are associated with r=0.95 (the probability) and r=0.90 (the average number of HBs). The dashed black and red lines in (**c**) correspond to r=−0.98 (probability) and r=−0.99 (the average number of HBs).

The water orientation plots (see Section 2.2. for details) in Figure 5a show the average orientation of water in the hydration layer adjacent to the backbone atoms of the guest residue. The leftmost plot in Figure 5a shows three characteristic water orientations, defined relative to the SAS normal $\hat{n}$. Water orientations centered at ($\eta \approx 120^{\circ }$, $\theta \approx 90^{\circ }$) that correspond to one of the water hydrogens pointing toward the SAS dominate all four water orientation plots. We calculated the probability that hydration water adopts the most populated orientations within the black rectangle in Figure 5a (see Section 2.2. for the definition) and performed a linear regression analysis to test whether or not this probability correlates with the pPII, β (the sum of aβ and βt), and/or α-helical population. Our results demonstrate that this probability (Table S4, column 2) correlates with the pPII population (black solid line in Figure 5b with Pearson’s r=0.92) and is anti-correlated with the β population (black solid line in Figure 5c, r=−0.89). The repeated regression analysis with shifted pPII and β populations of the central glycine in GGG (corresponding to the shifted mesostate regions, see Table S2, and marked as open symbols in Figure 5b,c) revealed that this probability correlates with pPII and anti-correlates with β even better (black dashed lines in Figure 5b,c with the corresponding Pearson’s r=0.95 and r=−0.98, respectively). In contrast, no correlation between this probability and α-helical populations exists (black solid line in Figure S6, r=0.36).

Table S5 shows the average number of water-peptide HBs for each of the three residues of the four tripeptides. The average number of HBs between water and the guest residue in GGG, GAG, AAA, and GPG (Table S5, Residue 2) normalized by the number of functional groups (two for GGG, GAG, and AAA, and one for GPG) also correlates with the pPII population (r=0.86) and anti-correlations with the β population (r=−0.93). These correlations again improve (r=0.90 and r=−0.99, respectively) when the pPII and β propensities for the central glycine residue in GGG are replaced by their shifted values (Table S2). The average number of HBs between the water and guest residue does not correlate with the α helix propensity (red solid line in Figure S6, r=0.35). Tables S6 and S7 show the average number of HBs that water forms with each backbone amide group and carbonyl group, respectively. The results in Tables S6 and S7 demonstrate that the correlation between the pPII population of the guest residue and water-peptide HBs stems from the amide group of the guest residue. The ammonia hydrogens of residue 1 form the most HBs with water, followed by the amide hydrogen of the guest residue (proline excluded), whereas the amide hydrogen of residue 3 forms the least HBs with water (Table S6). The environment of the amide group can be experimentally probed by chemical shift measurements. Previously reported values for GAG [72,73] (Table S8) are consistent with an increased hydration of the amide group of residue 2 relative to residue 3 (Table S6, columns 3 and 4) as increased hydration produces upfield shift of the chemical shift [74]. The carbonyl oxygen of residue 1 forms significantly fewer HBs with water than carbonyl oxygens of residues 2 and 3 (Table S7). The rather large intrinsic wavenumber differences between the two amide I’ bands in the spectra for the N- and C-terminal modes for GGG (Figure S1), GAG [19] and AAA [38] (Table S8) are consistent with stronger hydrogen bonding of the carbonyl oxygen of residue 2 relative to that of residue 1 to water because stronger hydrogen bonding causes a downshift of the amide wavenumber.

**Figure 6:**
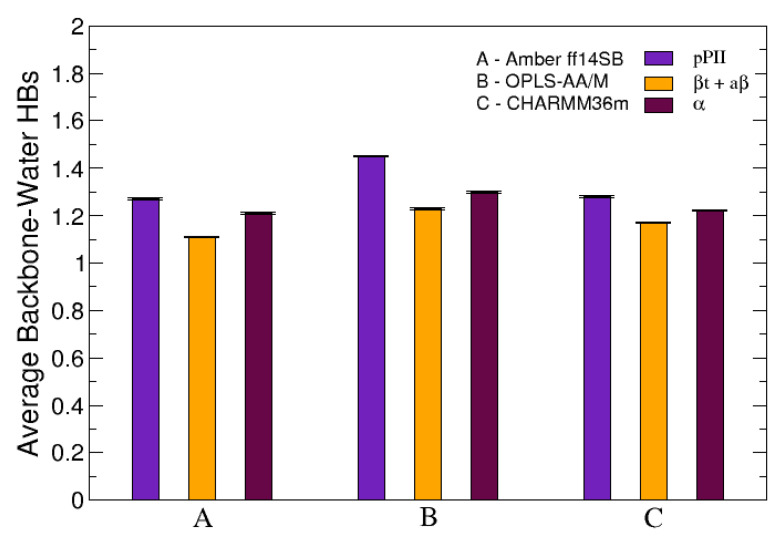
The average number of HBs between the functional backbone groups of the central glycine in GGG and water calculated individually for each of the three mesostates: pPII, β-strand (comprising both aβ and βt as defined in Section 2.2.) and right-handed helical (α). The error bars correspond to the SEM values.

The average number of HBs was also investigated when the conformational ensemble of central glycine in GGG was separated into three groups corresponding to three mesostates (pPII, β-strand and α helix). Figure 6 demonstrates that, in all three force fields used in this study, pPII is the mesostate that maximizes the number of HBs between the functional backbone groups of guest glycine and water. These results are analogous to the results for guest alanine in GAG and AAA in our previous study [39]. Combined, these findings elucidate pPII as the mesostate that allows for the most HBs between water and functional groups of guest glycine and alanine.

### 3.4. DMSO Reduces PPII Content of the Central Glycine in GGG

Our findings above indicate that glycine’s tendency to adopt the pPII state might be driven by water’s tendency to form HBs with the functional groups of glycine residue. For a solvent with a reduced propensity for hydrogen bonding with functional peptide groups, we would thus expect the pPII content to decrease. To this end, we performed MD simulations of GGG in DMSO, a polar solvent with the ability to form HBs with the peptide limited to the NH groups. MD simulations of GGG in DMSO were conducted within Amber ff14SB and CHARMM36m to obtain the Ramachandran distributions shown in Figure S7a,b. The respective mesostate populations are reported in Table S2. It is worth noting that the two force fields produced significantly different Ramachandran distributions. In comparison to CHARMM36m, Amber ff14SB predicts a much higher preference for right-handed helical conformations and also produces the Ramachandran distribution which more strongly deviates from the respective distribution in water. The changes in the β-strand population induced by replacing water by DMSO also depended on the force field, whereby β-strand populations were diminished in Amber ff14SB but slightly increased in CHARMM36m. Regardless of the force-field dependent features of the Ramachandran distributions, both force fields predict strongly reduced pPII populations. When water was replaced by DMSO in Amber ff14SB, the pPII population of central glycine in GGG was reduced four-fold, from 0.36 to 0.09 (using the original definition of the pPII mesostate) or from 0.42 to 0.10 (using the definition of the pPII mesostate that is shifted by 31${}^{\circ }$ in the positive ψ-direction). In CHARMM36m, replacing water by DMSO caused a reduction of the pPII population from 0.48 to 0.27 for the original pPII mesotate definition or from 0.72 to 0.37 for the shifted pPII mesostate; in either case, the change was close to a factor of 2. The average number of HBs between the guest glycine backbone and DMSO was 0.26 ± 0.007 and 0.51 ± 0.015 in Amber ff14SB and CHARMM36m, respectively. Thus, in Amber ff14SB, DMSO exhibits a lower affinity for hydrogen bonding with glycine residue when compared to DMSO in CHARMM36m, which may explain why replacing water by DMSO exerts a larger reduction in the pPII population in the former. Thus, in both force fields, pPII content of central glycine in GGG is significantly diminished when water is replaced by a solvent with a limited ability for HB formation, and this reduction correlates with the degree to which DMSO forms HBs with the backbone functional groups of glycine residue. Although there is no experimental data on conformational ensemble of central glycine in GGG, Eker et al. reported that, while alanine-based tripeptides favor pPII in water, their pPII content is significantly reduced in DMSO [75]. Consistent with the key role of water in pPII stabilization, the addition of ethanol to water was also shown to disfavor the pPII mesostate of guest alanine in cationic GAG [72,76].

### 3.5. Nonpolar Solvent Further Reduces PPII Content of the Central Glycine in GGG

As shown above, limited capacity of DMSO to form HBs with functional backbone groups of glycine results in a decreased pPII population. To examine what happens if the solvent cannot form HBs, we performed MD simulations of GGG in a nonpolar solvent, CCl${}_{4}$, using Amber ff14SB and CHARMM36m. The Ramachandran distributions of the central glycine in GGG shown in Figure S7c,d reveal that both force fields reflect the conformational landscape of glycine in the gas phase fairly well [71]. The populations of the four mesostates of interest defined in Section 2.2. are reported in Table S2. The most notable feature of the Ramachandran distributions for glycine residue in CCl${}_{4}$ is the strong preference for 2${}_{7}$ helical conformations that are absent from the Ramachandran distribution of glycine residue in water. Both MD force fields result in a large population within the 2${}_{7}$ helical region, which may be in part stabilized by intrapeptide HBs. The propensities for intrapeptide HB formation are 0.34 and 0.04 for Amber ff14SB and CHARMM36m, respectively (Table S9, Column 3). The 2${}_{7}$-helical conformation of the central glycine is associated with a HB between the CO group of the N terminal glycine and the NH group of the C terminal glycine. When only these functional groups are considered, the propensity for the HB formation is 0.09 and 0.04 for Amber ff14SB and CHARMM36m, respectively (Table S9, Column 1). In CHARMM36m, the intrapeptide HB between the above functional groups is the only HB that can form in the system. In Amber ff14SB, the neutrally capped N- and C-terminal groups can also be involved in HB formation. Regardless of the force field, the intrapeptide HB propensity alone is not sufficiently high to explain why the central glycine in GGG prefers the 2${}_{7}$-helical over other conformations when embedded into a nonpolar solvent, suggesting that factors other than hydrogen bonding drive this conformational preference. Importantly, both MD force fields result in a strongly diminished pPII mesostate population. While the interpretation of the data obtained in MD simulations in CCl${}_{4}$ may be complicated by the fact that in this solvent the N-terminal capping of GGG differs from the positively charged N terminus in water and DMSO, the comparison of Ramachandran distributions in DMSO and CCl${}_{4}$ is nonetheless revealing. In Amber ff14SB, the pPII population increases when DMSO is replaced by CCl${}_{4}$ from 0.09 to 0.18, but this is due to a relatively small population that occupies the lower part of the original pPII region (Figure S7a,c). If the shifted pPII region is used, then the pPII population decreases from 0.10 to 0.02 (Table S2). Table S2 also shows that, in CHARMM36m, the pPII population of the central glycine in GGG decreases when DMSO is replaced by CCl${}_{4}$: from 0.27 to 0.13 for the original pPII region and, even more so, from 0.37 to 0.09 for the shifted pPII region. This trend of diminishing pPII populations with decreased backbone-solvent hydrogen bonding is consistent with our hydration analysis and showcases the critical role of water in stabilizing the pPII state.

## 4. Discussion

The goal of the current study is to determine the intrinsic conformational propensity of the peptide or protein backbone in water in terms of the backbone dihedral angles ϕ and ψ. To this end, we investigated the conformational distribution of the central glycine residue in cationic GGG. We selected this oligo-glycine peptide for three reasons. First, the absence of a heavy-atom side chain in the central glycine ensures that its conformational ensemble will not be affected by direct backbone-side chain interactions. Second, the choice of neighboring glycines minimizes the effect of side-chain–side-chain effects among nearest neighbors. Third, experimental data (amide I’ profiles and J-coupling constants) for this peptide are already available. The analysis of these spectroscopic data described here invokes the Gaussian model, which describes the Ramachandran distribution of the central glycine in GGG as a linear combination of statistically weighted two-dimensional Gaussian sub-distributions, optimized to best fit the experimental data, resulting in the first experiment-based evidence that the Ramachandran space of glycine residue in water is dominated by the pPII state. A comparison of Gaussian Ramachandran distributions of the central glycine residue in GGG and alanine residue in GAG [39] demonstrates that the restricted conformational space of glycine is comparable to that of alanine except that alanine is chiral and, therefore, the conformations of the right part of its Ramachandran map are less accessible. This supports the notion that the high propensity of residue for pPII, which has been found to be particularly pronounced for alanine [9,10,11,12,13,14], reflects the peptide backbone properties and its affinity to form HBs with water rather than the peculiar nature of its side chain.

We further demonstrate that MD simulations with three commonly used MD force fields, namely Amber ff14SB, OPLS-AA/M, and CHARMM36m, do not fully capture spectroscopic data for the central glycine in cationic GGG. Nonetheless, the Ramachandran distributions obtained with all three force fields reproduce the upshift of the pPII basin (relative to that of alanine in GAG [39]) and the dominance of pPII over β-strand populations, in agreement with the Gaussian Ramachandran distribution. The main difference between MD-derived and Gaussian Ramachandran distributions is the relative populations of pPII and right-handed helical populations, whereby all MD simulations overestimate the latter. In agreement with several earlier studies, the analysis of peptide-hydration produced by the MD simulations reveals that the conformational preference for pPII is driven by water whereby pPII emerges as the mesostate that allows glycine residue to optimize hydrogen bonding with water. Replacing water by organic solvents with limited ability or inability to form HBs with the peptide backbone further demonstrates the direct relationship between backbone-solvent HBs and the pPII mesostate populations. The role of peptide/protein–water interaction as a stabilizer of pPII has been emphasized in many earlier publications [10,25,27,77,78,79,80,81,82,83]. Therefore, the question arises how the findings of this study fit into a broader understanding of how interactions of backbone and side chain groups with water control intrinsic conformational propensities of amino acid residues. With respect to pPII stabilization, three aspects of peptide-water interactions are discussed in the literature. As first suggested by Garcia [78] and later modified by Osman, Rose, and collaborators [80,84,85], pPII facilitates backbone hydration and minimizes the differences between the structure of hydration and bulk water. Amino acid side chain groups modify backbone hydration, specifically the amide groups. The influence of side chains has been quantified by Fleming et al. in terms of so-called conditional hydrophobic accessible surface area of side chains (CHASA) [80]. By implementing a concept of conditional solvation as introduced by Ben-Naim [86], CHASA values were calculated after first positioning water molecules at hydrogen bonding distances from amide and carbonyl groups. This study found that CHASA values varied significantly between pPII and β-strand conformations of respective residues and the resulting pPII preferences exhibited medium level correlation with pPII propensities inferred from coil libraries [80]. However, due to involvement of hydrophobic side chain-solvent interactions, the stabilization of pPII was predicted to be entropic in nature. Based on MD simulations of oligo-alanines in water, Mezei et al. corroborated this notion by claiming a higher ordering of water in the hydration shell of β-strand conformations, suggesting entropic nature of pPII stabilization [85]. This conclusion is invalidated by the experimental results of Toal et al., who demonstrated that the pPII conformations of guest residues x in GxG and central alanine in AAA are stabilized enthalpically [87]. MD-data based findings in this work reveal the importance of water-residue hydrogen bonding in stabilization of pPII conformations, which is consistent with experimental data [20,87]. Another study reported that pPII stabilization arises from screening of electrostatic interactions between the functional groups of adjacent peptide groups [27]. The screening prevents the backbone from adopting the more extended structure of the gas phase (if one ignores intramolecular hydrogen bonding). It is reasonable to surmise that such screening effects would add to the solvation energy of the peptide backbone. Avbelj et al. showed that distinct side chains modulate the solvation energy such that bulky, aliphatic side chains prefer β-strand over pPII conformations [27]. None of the above pPII stabilization hypotheses provide much information about the role of hydrogen bonding between water and backbone groups, which is elucidated in this work. Our findings are consistent with the results of two DFT studies [81,88]. Ilawe et al. investigated cationic GxG peptides in implicit and explicit water (10 water molecules) reporting that an explicit consideration of interaction between water and peptide groups is needed for pPII stabilization [81]. Using DFT calculations on zwitterionic trialanine, Lanza and Chiachio showed that, without explicit consideration of water molecules, hydrogen bonded to backbone groups the pPII conformation is insufficiently stabilized [88]. These DFT calculations used AAA in a bath of explicit water, whereby increasing number of water molecules (up to 41) was added, to show that the pPII state is stabilized by dipole–dipole interactions between the respective peptide groups and hydrogen bonded water [88]. The results of our study add two important pieces of information to the understanding of pPII stabilization. First, by demonstrating the high pPII propensity of glycine residue in water, we corroborate the notion that backbone solvation is the main contributor to pPII stabilization. Second, we show that the pPII population of glycine residue in cationic GGG correlates with the number of water-backbone HBs, which is consistent with our previous study reporting that the pPII mesostate maximizes the number of HBs between water and the backbone of the central alanine in GAG and AAA [39]. Our current results thus support the notion of the enthalpically favored and entropically disfavored pPII state [87], in which water molecules are more ordered due to preferential hydrogen bonding to the functional backbone groups. This result implies that the methyl side chain of alanine residue causes a minimal disturbance of the backbone solvation by accommodating water molecules through formation of clathrate water structure around it [25]. We thereby posit that residues with more sterically demanding side chains could significantly perturb the hydration layer and thereby affect the ability of water to form HBs with the functional backbone groups. How the interplay between distinct heavy-atom side chains and backbone hydration affects the intrinsic conformational ensembles of amino acid residues in water is still an open question.

Finally, glycine residues are typically associated with turn formation in proteins and are expected to increase local flexibility in proteins. Our comparison of Shannon entropy associated with Ramachandran distributions of glycine and alanine residues in GGG and GAG, respectively, reveals a surprisingly low entropy associated with glycine relative to that of alanine, suggesting that glycine might be a lot less flexible when embedded into a chiral environment of a protein than previously expected. The high intrinsic pPII propensity of glycine might elucidate the mechanism by which polyglycine forms a pPII-type 3${}_{1}$-helix in the solid state [89] and maintains this structure in aqueous solution, even at a high ion concentration [90]. Our findings are relevant to formation of pPII helices in collagen, in which glycine alternates with proline [91]. Moreover, Gates et al. reported that the snow flea antifreeze protein adopts a stable fold without hydrophobic core, α helix, or β sheet formation [92]. Instead, this protein with 46 glycine residues forms stable bundles of pPII helices connected by HBs. The authors attributed this peculiar fold to the high level of pPII dihedral angle bias [92], which is consistent with findings reported here.

## Supplementary Materials

The following are available online at https://www.mdpi.com/2218-273X/10/8/1121/s1, Table S1: Experimental and calculated J-coupling constants, Table S2: Mesostate populations, Table S3: Shannon entropy differences, Table S4: Hydration layer characteristics, Table S5–S7: Hydrogen bonding analysis, Table S8: Chemical shift measurements and intrinsic wave number differences between two amide I’ bands, Table S9: Intrapeptide hydrogen bonding analysis for simulations in CCl${}_{4}$, Figure S1: Amide I’ profiles, Figure S2: MD-derived J-coupling constants versus simulation time, Figure S3: Amide I’ VCD signals for different sampling times, Figure S4: Ramachandran distributions with different C-terminal groups, Figure S5: Calculated J-coupling constants and VCD signals for different C-terminal groups, Figure S6: Relationship of water orientation probability and average number of HBs to α helical populations, Figure S7: Ramachandran distributions of central glycine of GGG in DMSO and CCl${}_{4}$.

## Abbreviations

The following abbreviations are used in this manuscript:

MD	Molecular Dynamics
SAS	Solvent Accessible Surface
HB	Hydrogen Bond
pPII	polyproline II
