# Supplementary material for: Glycine in Water Favors the Polyproline II State
Source: Biomolecules. 2020 Jul 29;10(8):1121. doi: 10.3390/biom10081121 (PMC7463814; doi:10.3390/biom10081121)
Supplement: Supplementary file 1 [file biomolecules-10-01121-s001.pdf]

Supplementary Materials: Glycine in Water Favors the Polyproline II State

Brian Andrews [1], Shuting Zhang [1], Reinhard Schweitzer-Stenner[2] and Brigita Urbanc [1]*

Supplementary Results

The experimental study by Toal *et al.* demonstrated that the charges and the physico-chemical properties of the C-terminal capping of GAG, alanine dipeptide, and AAA do not significantly affect the conformational ensembles of the guest alanine residue [S1]. This study also revealed that the conformational distributions of alanine in GAG and alanine dipeptide are practically identical as a further demonstration that the C-terminal capping does not affect the conformational ensembles of the guest residues in these short peptides [S1]. We here revisit the question to which extent the C-terminal capping of GGG affects the Ramachandran distribution and reproduction of spectroscopic data of the guest glycine in GGG in the three MD force fields under study. The negatively charged C-terminus in all three MD force fields are the same (COO^-). As the neutral C-terminal capping of GGG, COOH is used in OPLS-AA/M and CHARMM36m, whereas in Amber ff14SB CO-NH_2 is the only choice for the neural C-terminal capping. The Ramachandran distributions comparing the two C-terminal groups of each force field are shown in Fig. S3, whereas the corresponding J-coupling constants and VCD amide I′ profiles are displayed Fig. S4. These results combined indicate minor effects induced by negative versus neutral C-terminal groups. The exception is the VCD signal derived from the Ramachandran distribution of glycine obtained from OPLS-AA/M simulations, which increases by a factor of ten for central glycine in GGG with a negative C-terminal group relative to GGG with the neutral capping. Despite this tenfold increase, the VCD amide I′ signal remains below the experimentally detectable value.

Supplementary Tables

Table S1. Experimental and calculated J-coupling constants and the uncertainty values used in χ^2_J calculations for the guest glycine in GGG. The MD-derived values for each of the three force fields are based on conformations within 50–300 ns of each trajectory.

	$^3J(H^N, H^{C_\alpha})$	$^3J(H^N, C')$	$^3J(H^{C_\alpha}, C')$	$^3J(C, C')$	$^1J(N, C_\alpha)$
Experimental	5.89	1.10	4.01	0.26	12.17
Gaussian	5.94	1.16	3.95	0.66	11.78
Amber ff14SB	5.97	1.14	3.55	0.99	11.40
OPLS-AA/M	6.01	1.05	3.25	1.25	11.60
CHARMM36m	5.99	1.17	3.93	0.60	11.69
Uncertainty	0.02	0.07	0.1	0.03	0.07

Table S2. Mesostate populations of central residues in GGG, GAG, AAA, and GPG. For the central glycine in GGG, the populations based on the original mesostate definitions given in *Methods* and shifted pPII and β populations (centers of the two basins shifted by 31° and 16°, respectively, in the $+\psi$ direction) are shown.

Populations	pPII	βt	aβ	α
**GGG in water (original/shifted)[a]**				
**Gaussian model**	0.46/0.60	0.13/0.17	0.01/0.03	0.06
**Amber ff14SB**	0.36/0.42	0.05/0.07	0.09/0.12	0.05
**OPLS-AA/M**	0.27/0.30	0.15/0.17	0.13/0.18	0.02
**CHARMM36m**	0.48/0.72	0.02/0.04	0.01/0.01	0.04
**GAG in water[b]**				
**Gaussian model**	0.59	0.16	0.02	0.02
**Amber ff14SB**	0.55	0.07	0.13	0.09
**OPLS-AA/M**	0.48	0.15	0.11	0.03
**CHARMM36m**	0.55	0.09	0.12	0.06
**AAA in water[b]**				
**Gaussian model**	0.76	0.09	0.03	0.03
**Amber ff14SB**	0.63	0.07	0.10	0.07
**OPLS-AA/M**	0.55	0.18	0.10	0.02
**CHARMM36m**	0.57	0.09	0.11	0.04
**GPG in water**				
**Amber ff14SB**	0.82	0.01	0	0.08
**OPLS-AA/L[c]**	0.86	0.01	0	0.03
**CHARMM36m**	0.62	0.01	0	0.05
**GGG in DMSO[a]**				
**Amber ff14SB**	0.09/0.10	0.02/0.03	0.05/0.06	0.18
**CHARMM36m**	0.27/0.37	0.04/0.06	0.01/0.02	0.08
**GGG in CCl_4[a]**				
**Amber ff14SB**	0.18/0.02	0/0	0.02/0.03	0
**CHARMM36m**	0.13/0.09	0.05/0.02	0.05/0.07	0.02

[a] Includes both right and left-handed mesostate populations.
[b] Data taken from Zhang *et aal.* [S2].
[c] Proline is currently not implemented in OPLS-AA/M.

Table S3. Shanon entropy differences ΔS_I and ΔS_{II}. The first two rows show the Shannon entropy difference between each of the three MD force fields and Gaussian model (ΔS_I) for the guest glycine in GGG and alanine in GAG. The last row shows the Shannon entropy difference between the guest glycine in GGG and alanine in GAG (ΔS_{II}) for the Gaussian model and the three MD force fields.

ΔS_I[J mol^{-1} K^{-1}]	Gaussian	Amber ff14SB	OPLS-AA/M	CHARMM36m
ΔS_{GGG}	–	4.90	5.07	-1.66
ΔS_{GAG}	–	0.66	1.49	-0.83
ΔS_{II}[J mol^{-1} K^{-1}]	Gaussian	Amber ff14SB	OPLS-AA/M	CHARMM36m
S_{GGG} - S_{GAG}	3.24	7.48	6.81	2.41

Table S4. Characterization of the hydration layer adjacent to the backbone of the guest residue in GGG, GAG, AAA, and GPG. The table shows the probability of the most populated water orientations, the average number of water molecules (H_2Os), and the average number of H_2Os per unit SASA and their respective SEM values. The backbone hydration layer SASA values are 1.96×10^{-3} nm^2 for guest residues in GGG, GAG, and AAA. The corresponding SASA value for proline in GPG is 1.90×10^{-3} nm^2.

	Water Orientation Probability	Number of H_2Os	Number of H_2Os per unit SASA [nm^{-2}]
GGG	$0.139 \pm 7 \times 10^{-4}$	12.3 ± 0.20	6.27
GAG	$0.142 \pm 6 \times 10^{-4}$	14.3 ± 0.02	7.30
AAA	$0.149 \pm 7 \times 10^{-4}$	13.4 ± 0.01	6.83
GPG	$0.171 \pm 8 \times 10^{-4}$	9.3 ± 0.40	4.89

Table S5. The average number of water-peptide HBs for GGG, GAG, AAA, and GPG derived from MD simulations with Amber ff14SB. The error bars correspond to SEM values.

	Residue 1	Residue 2	Residue 3	Total
GGG	1.20 ± 0.006	1.19 ± 0.006	1.19 ± 0.006	3.62 ± 0.007
GAG	1.19 ± 0.006	1.27 ± 0.004	1.19 ± 0.006	3.67 ± 0.009
AAA	1.45 ± 0.007	1.30 ± 0.006	1.25 ± 0.007	4.01 ± 0.010
GPG	1.12 ± 0.006	1.08 ± 0.004	1.21 ± 0.006	3.41 ± 0.010

Table S6. The average number of HBs between water oxygens and amide hydrogens in GGG, GAG, AAA, GPG derived from MD simulations with Amber ff14SB. The error bars correspond to SEM values.

	NH of Residue 1[a]	NH of Residue 2	NH of Residue 3	All NH Groups
GGG	0.56 ± 0.004	0.23 ± 0.003	0.19 ± 0.003	0.99 ± 0.006
GAG	0.56 ± 0.004	0.26 ± 0.003	0.20 ± 0.003	1.02 ± 0.007
AAA	0.84 ± 0.006	0.33 ± 0.003	0.23 ± 0.004	1.40 ± 0.007
GPG	0.56 ± 0.004	-	0.21 ± 0.004	0.78 ± 0.006

[a] N-terminal NH_3^+.

Table S7. The average number of HBs between water hydrogens and carbonyl oxygens in GGG, GAG, AAA, and GPG derived from MD simulations with Amber ff14SB. The error bars correspond to SEM values.

	CO Residue 1	CO Residue 2	CO Residue 3[a]	All CO Groups
GGG	0.64 ± 0.004	0.98 ± 0.004	1.01 ± 0.006	2.63 ± 0.007
GAG	0.63 ± 0.004	1.01 ± 0.004	1.00 ± 0.004	2.64 ± 0.008
AAA	0.61 ± 0.004	0.98 ± 0.004	1.03 ± 0.004	2.62 ± 0.008
GPG	0.56 ± 0.004	1.08 ± 0.004	1.00 ± 0.004	2.64 ± 0.007

[a] C-terminal $CONH_2$.

Table S8. Chemical shift measurements for GAG [S3] and AAA [S4] probing the amide groups of residues 2 and 3 (columns 2 and 3). Intrinsic wavenumber differences between two amide I′ bands (the N- and C-terminal modes) in the spectra of GGG (Fig. S2), GAG [S5], and AAA [S6] (columns 4 and 5).

Residue	Chemical Shift NH Residue 2 [ppm]	Chemical Shift NH Residue 3 [ppm]	Amide I′ Wavenumber (Residue 1) [cm^{-1}]	Amide I′ Wavenumber (Residue 2) [cm^{-1}]
GGG	-	-	1681	1659
GAG	8.42	8.58	1676	1654
AAA	8.49	8.58	1676	1652

Table S9. The propensity for intrapeptide HB formation in GGG in nonpolar CCl_4. The first two columns correspond to specific HBs associated with the 2_7 and 3_{10} helices. The groups involved in HB formation are in parentheses and subscripts 1 and 3 refer to the N-terminal and C-terminal glycines in GGG, respectively. The third column corresponds to the total intrapeptide HB propensity regardless of the groups involved in hydrogen bonding. The error bars correspond to SEM values.

	$(CO)_1$-$(NH)_3$ 2_7 Helix	$(NH_2)_1$-$(CONH_2)_3$ or $(NH_2)_1$-$(COOH)_3$ 3_{10} Helix	Total
Amber ff14SB	0.093 ± 0.003	0.004 ± 0.0005	0.340 ± 0.006
CHARMM36m	0.040 ± 0.001	< 0.001	0.041 ± 0.001

21 ## Supplementary Figures

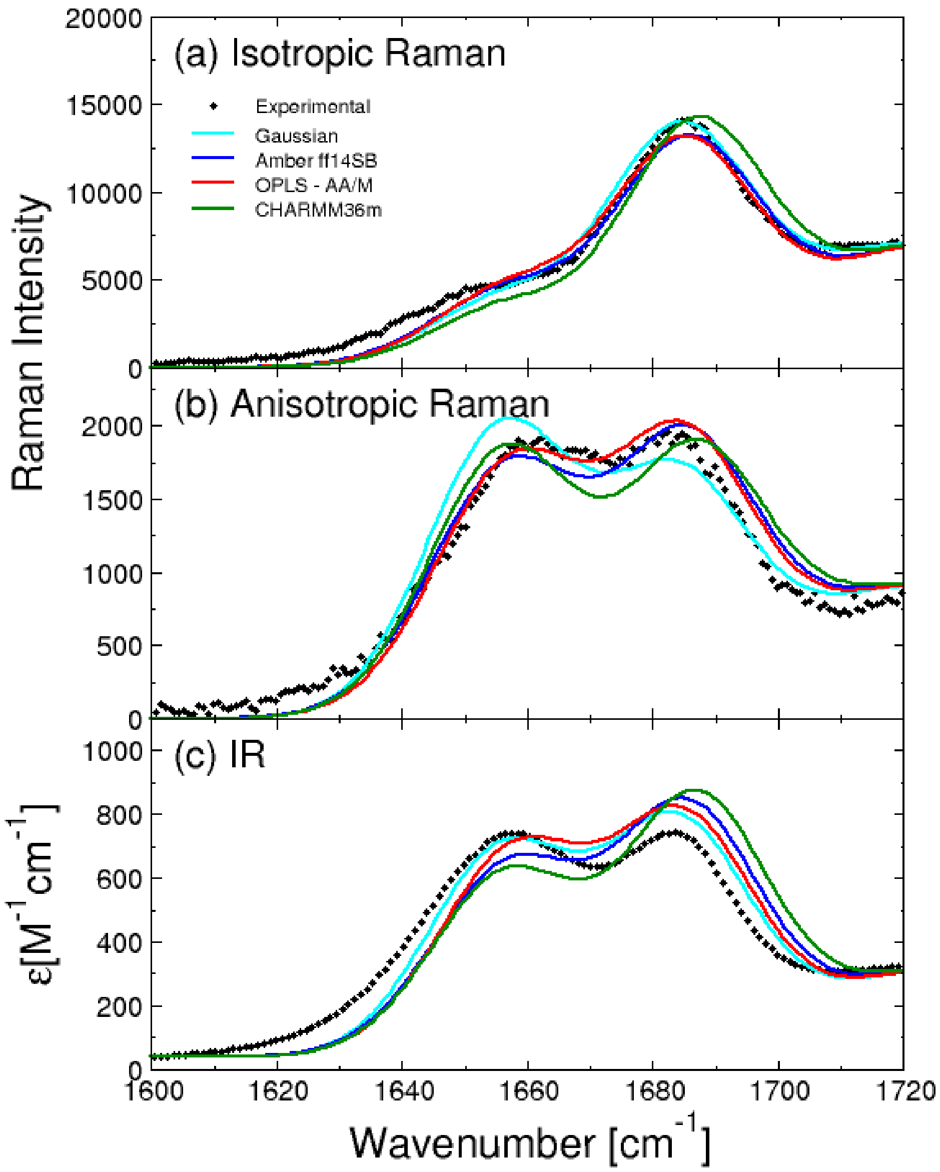

Figure S1. Amide I′ profiles for the central glycine in GGG. Experimental amide I′ profiles derived from (a) isotropic Raman, (b) anisotropic Raman, and (c) IR spectroscopy measurements are compared to predictions of the Gaussian model and MD simulations with Amber ff14SB, OPLS-AA/M, and CHARMM36m.

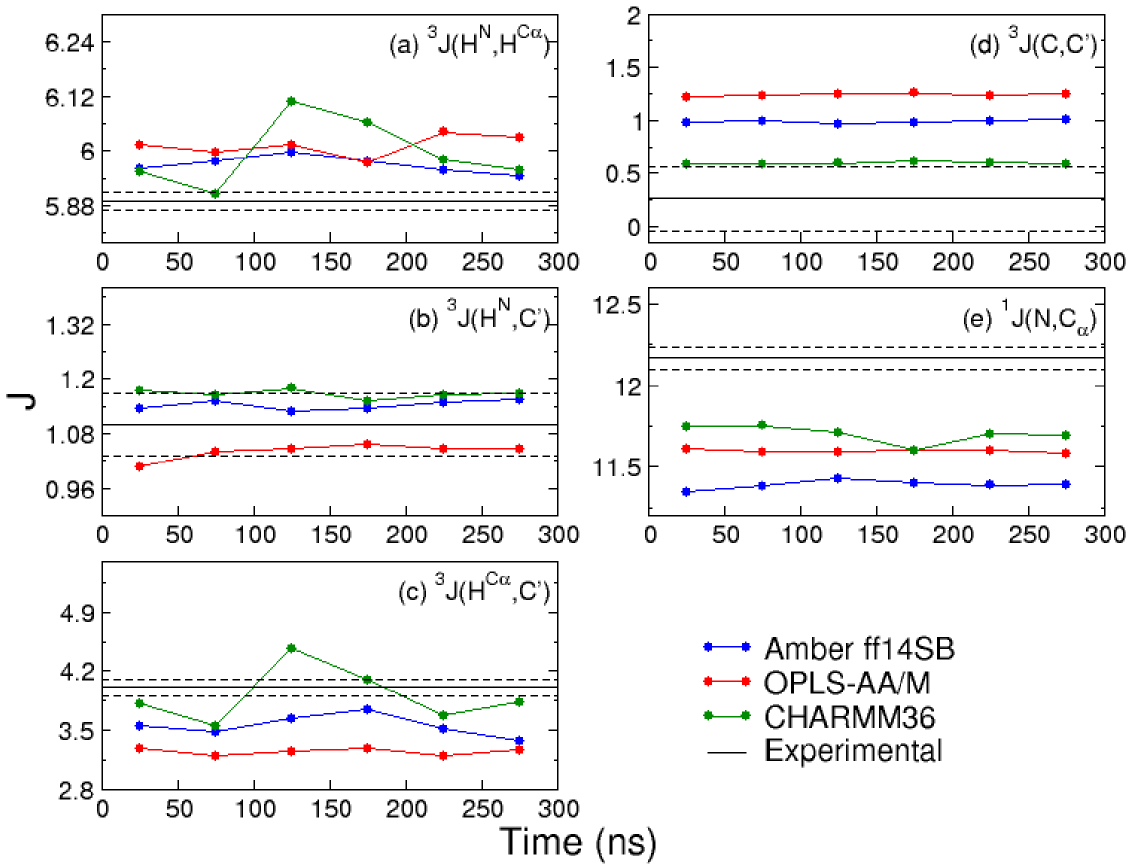

Figure S2. Five J-coupling constants corresponding to the central glycine in GGG calculated for successive 50 ns–long intervals along 300 ns– long MD trajectories for each of the three force fields. Solid and dashed lines correspond to experimental values and the respective uncertainties.

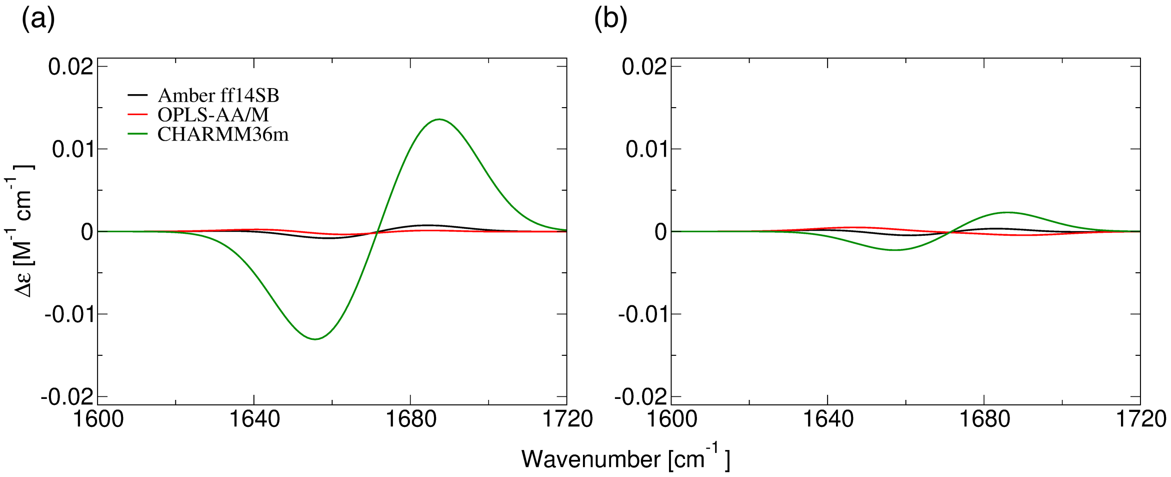

Figure S3. VCD profiles for the central glycine in GGG calculated from Amber ff19SB, OPLS-AA/M, and CHARMM36m simulations by using data within (a) 50-100 ns and (b) 50-300 ns of each trajectory.

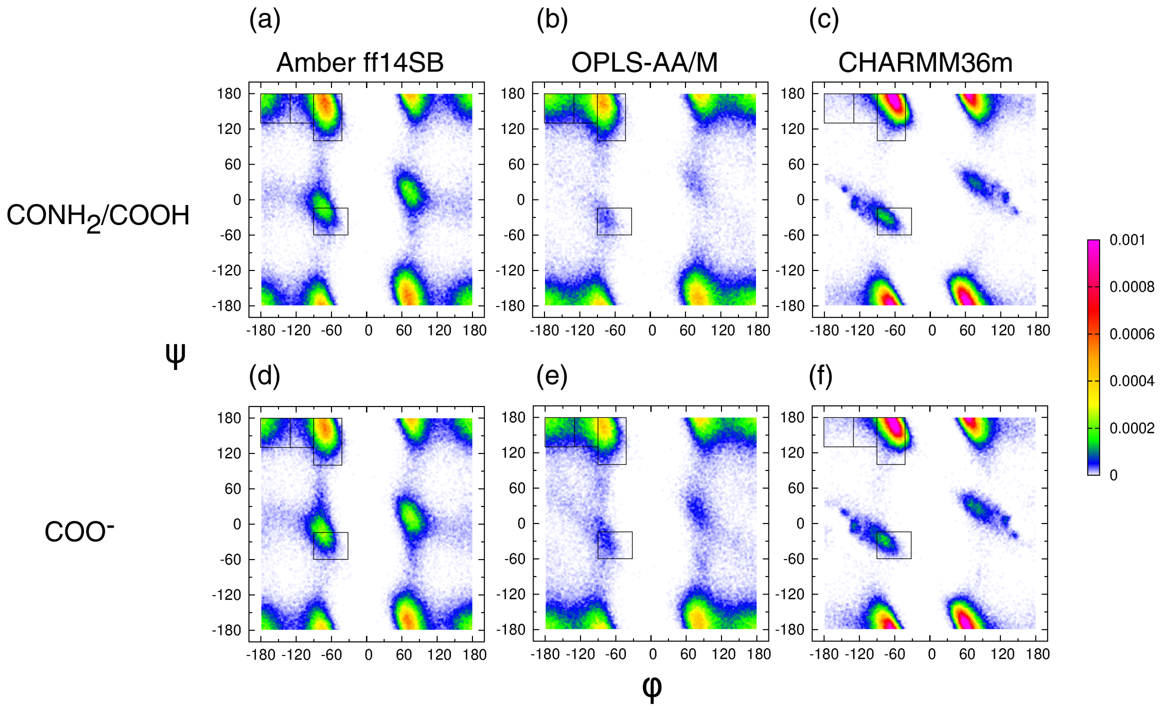

Figure S4. Comparison between Ramachandran distributions of the central glycine in cationic GGG with (a-c) neutral and (d-f) charged C termini. The rectangular boxes correspond to the four mesostates defined in *Methods* and Fig. 1a.

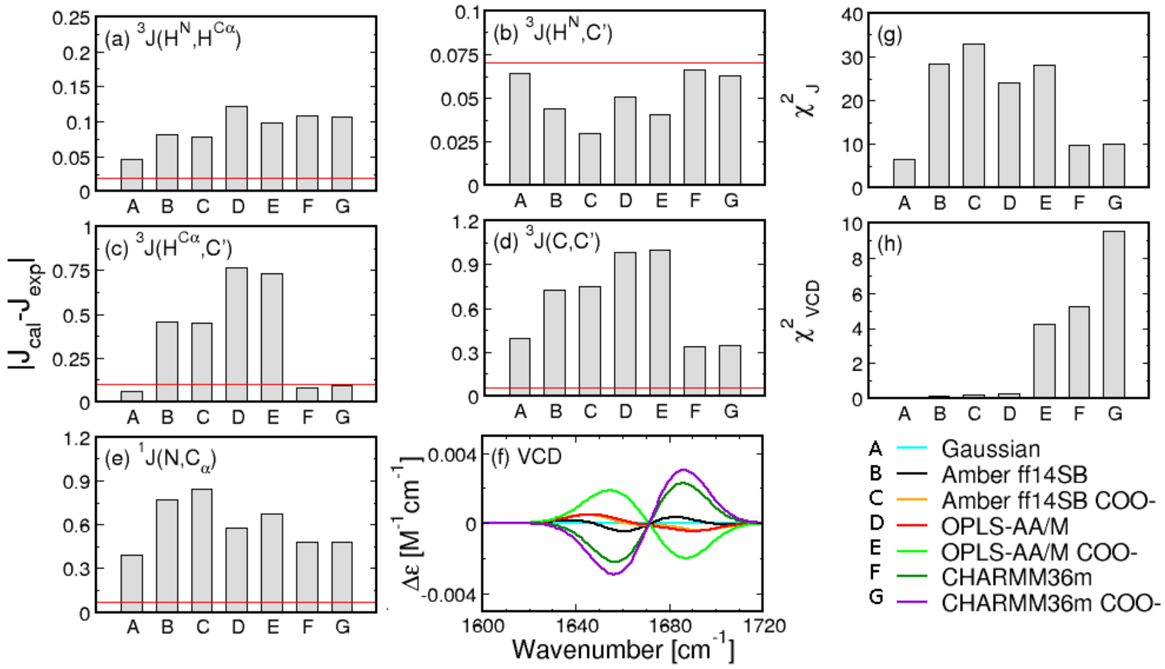

Figure S5. A comparison between experimental and calculated J-coupling constants and amide I′ profiles of the Gaussian model and three MD force fields probing the effect of the C-terminal capping of GGG on the conformational ensemble of the central glycine. (a-e) Absolute differences between calculated and experimental values of the five J-coupling constants for the Gaussian model and the three MD force fields. Red lines correspond to experimental uncertainties. (f) Amide I′ profiles calculated from MD-derived Ramachandran distributions. Note that the scale of the VCD profiles is adjusted from Fig. 2 to better highlight the differences. (g,h) The two χ^2 functions where χ^2_{VCD} values in (h) are multiplied by 10^7 for display purpose.

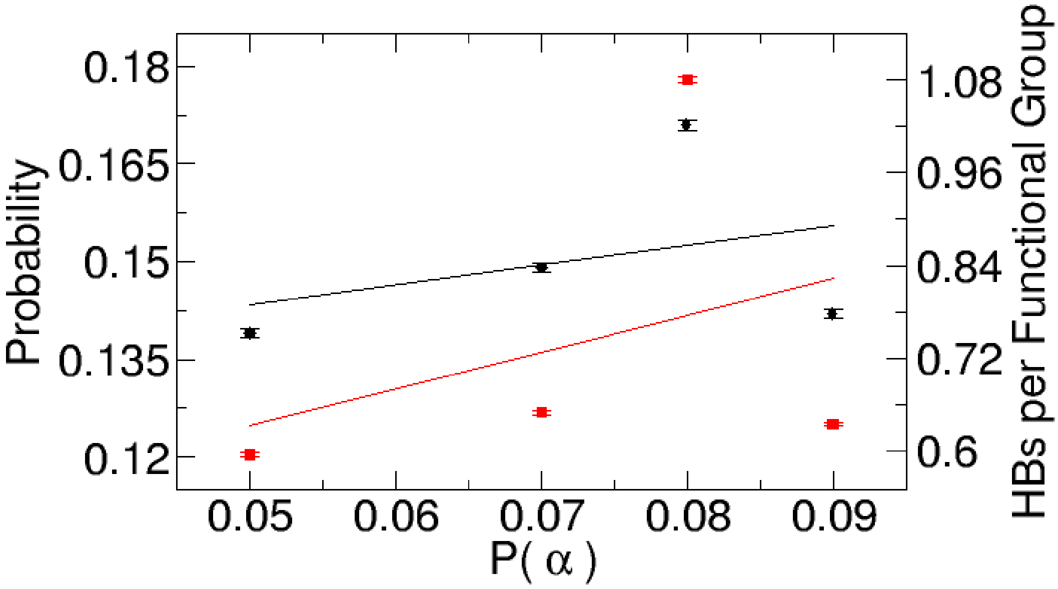

Figure S6. The probability of the most populated water orientations (left scale) and the average number of water-peptide HBs per functional group (right scale) as functions of the α-helical population of the guest residue in GGG, GAG, AAA, and GPG as derived by MD simulations with Amber ff14SB. Black and red lines are results of the linear regression analysis with the Pearson coefficients $r = 0.36$ and $r = 0.35$, respectively.

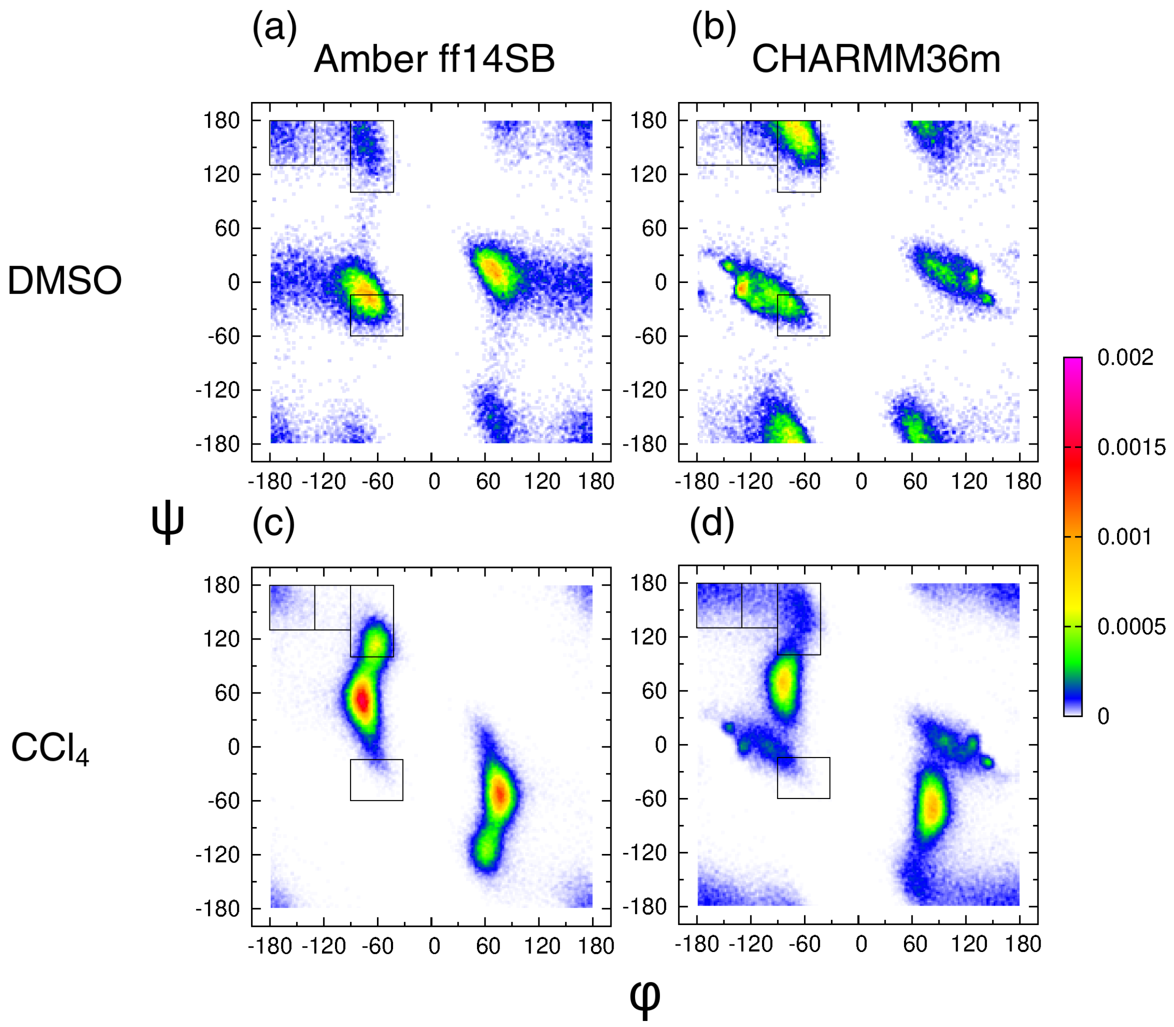

Figure S7. Ramachandran distributions of the central glycine in GGG derived from MD simulations in (a-b) DMSO and (c-d) CCl_4 within (a,c) Amber ff14SB and (b,d) CHARMM36m. The rectangular boxes correspond to the four mesostates defined in *Methods* and Fig. 1a.

References

[S1] Toal, S.; Meral, D.; Verbaro, D.; Urbanc, B.; Schweitzer-Stenner, R. The pH-independence of trialanine and the effects of termini blocking in short peptides: A combined vibrational, NMR, UVCD, and molecular dynamics study. *J. Phys. Chem. B* **2013**, *117*, 3689–3706.

[S2] Zhang, S.; Schweitzer-Stenner, R.; Urbanc, B. Do molecular dynamics force fields capture conformational dynamics of alanine in water? *J. Chem. Theory Comput.* **2020**, *16*, 510–527.

[S3] DiGuiseppi, D.; Milorey, B.; Lewis, G.; Kubatova, N.; Farrell, S.; Schwalbe, H.; Schweitzer-Stenner, R. Probing the conformation-dependent preferential binding of ethanol to cationic glycylalanylglycine in water/ethanol by vibrational and NMR spectroscopy. *J. Phys. Chem. B* **2017**, *121*, 5744–5758.

[S4] Toal, S.; Amidi, O.; Schweitzer-Stenner, R. Conformational Changes of Trialanine Induced by Direct Interactions between Alanine Residues and Alcohols in Binary Mixtures of Water with Glycerol and Ethanol. *J. Am. Chem. Soc.* **2011**, *133*, 12728–12739.

[S5] Hagarman, A.; Measey, T.J.; Mathieu, D.; Schwalbe, H.; Schweitzer-Stenner, R. Intrinsic Propensities of Amino Acid Residues in GxG Peptides Inferred from Amide I′ Band Profiles and NMR Scalar Coupling Constants. *J. Am. Chem. Soc.* **2010**, *132*, 540–551.

[S6] Schweitzer-Stenner, R.; Eker, F.; Huang, Q.; Griebenow, K. Dihedral angles of trialanine in D_2O determined by combining FTIR and polarized visible Raman spectroscopy. *J. Am. Chem. Soc.* **2001**, *123*, 9628–9633.
